# Understanding GPs’ referral decisions for younger patients with symptoms of cancer: a qualitative interview study

**DOI:** 10.3399/BJGP.2023.0304

**Published:** 2024-04-30

**Authors:** Erica di Martino, Stephanie Honey, Stephen H Bradley, Omer M Ali, Richard D Neal, Suzanne E Scott

**Affiliations:** Division of Primary Care, Public Health and Palliative Care, Leeds Institute of Health Sciences, University of Leeds.; Division of Primary Care, Public Health and Palliative Care, Leeds Institute of Health Sciences, University of Leeds.; Division of Primary Care, Public Health and Palliative Care, Leeds Institute of Health Sciences, University of Leeds.; Division of Primary Care, Public Health and Palliative Care, Leeds Institute of Health Sciences, University of Leeds.; Department of Health and Community Sciences, Faculty of Health and Life Sciences, University of Exeter.; Wolfson Institute of Population Health, Queen Mary University of London, London.

**Keywords:** age, cancer, general practice, guidelines, referral

## Abstract

**Background:**

Cancer incidence increases with age, so some clinical guidelines include patient age as one of the criteria used to decide whether a patient should be referred through the urgent suspected cancer (USC) pathway. Little is known about how strictly GPs adhere to these age criteria and what factors might influence their referral decisions for younger patients.

**Aim:**

To understand GPs’ clinical decision making for younger patients with concerning symptoms who do not meet the age criteria for USC referral.

**Design and setting:**

Qualitative study using in-depth, semi-structured interviews with GPs working in surgeries across England.

**Method:**

Participants (*n* = 23) were asked to recall consultations with younger patients with cancer symptoms, describe factors influencing their clinical decisions, and discuss their overall attitude to age thresholds in cancer referral guidelines. A thematic analysis guided by the Framework approach was used to identify recurring themes.

**Results:**

GPs’ decision making regarding younger patients was influenced by several factors, including personal experiences, patients’ views and behaviour, level of clinical concern, and ability to bypass system constraints. GPs weighted potential benefits and harms of a referral outside guidelines both on the patient and the health system. If clinical concern was high, GPs used their knowledge of local systems to ensure patients were investigated promptly even when not meeting the age criteria.

**Conclusion:**

While most GPs interpret age criteria flexibly and follow their own judgement and experience when making clinical decisions regarding younger patients, system constraints may be a barrier to timely investigation.

## Introduction

Cancer diagnosis in younger patients is challenging. The incidence of some malignancies, such as colorectal cancer, in younger patients is steadily increasing worldwide,^1^^–^3 with a 2023 study indicating a 28% rise in mortality due to early-onset cancer in 2019 compared with 1990.^3^ However, most types of cancers remain uncommon in people aged <50 years. Therefore, GPs are much more likely to suspect cancer in older patients,^4^ and younger patients may have to visit their GP three or more times before being referred for cancer tests.^5^^,^^6^ A delay in diagnosis could result in cancer progressing to a less curable stage. For some cancers, including colorectal cancer, there is evidence that younger patients are more likely to have advanced cancer at diagnosis, compared with patients in their 50s or 60s.^2^^,^^7^

When consulted by a patient with concerning symptoms, GPs have a range of investigative and referral options. In England, they can make an urgent suspected cancer (USC) referral (also called a 2-week-wait, relating to its target to be seen by a specialist within 2 weeks); they can refer for urgent investigations, such as colonoscopy, which will take a variable time depending on local provision; they can use a routine or non-urgent pathway; or they can investigate the patient in primary care, for example, using blood and stool tests. The National Institute for Health and Care Excellence (NICE) guidelines for suspected cancer often take age into account in referral criteria.^8^ For example, patients with unexplained rectal bleeding should be offered a USC referral if they are aged ≥50 years, but in younger patients this should only be considered if rectal bleeding is accompanied by other symptoms. Similarly, a USC referral is recommended for patients aged ≥30 years presenting with a breast lump, but a non-urgent referral is suggested for younger women.^8^

Consequently, clinical decisions for younger patients with symptoms of cancer may be challenging for GPs. Interestingly, a study has shown that the most common diagnostic route for patients <50 years with suspected colorectal cancer is through a non-urgent GP referral (33%), with only 22% diagnosed through the USC pathway, compared with 27–38% of people in older age groups.^9^

**Table:** How this fits in

Some cancers are becoming more common in younger people, yet clinical guidelines often recommend urgent referral for suspected cancer onlyif patients are above a certain age. Findings from this study show that, while most GPs interpret age criteria in cancer guidelines flexibly, some perceive and apply them as firm directives. In addition, system constraints may create unwarranted rigidity and act as barriers to prompt investigation. More in-built and explicit flexibility in the referral system is required to facilitate the timely diagnosis of younger patients perceived as at higher risk of cancer by their GP.

The aim of this study was to determine GPs’ views on age criteria in cancer referral guidelines, how strictly they apply them, and the influencing factors for their referral decisions for younger patients who they perceive to be at risk of having cancer. Throughout this article younger patients are referred to as those adults whose age is below the minimum age criteria for USC referral for a particular cancer.

## Method

### Design

This was a qualitative study using in -depth, semi-structured interviews with GPs working in England.

### Sample

Fully qualified GPs practising anywhere in England were eligible to take part. The recruitment strategy involved advertising through relevant clinical and research networks (for example, Cancer Alliances, West Yorkshire clinical commissioning groups, the Yorkshire and Humber Clinical Research Network, and the Cancer Research UK-funded CanTest Collaborative), social media, and the authors’ personal primary care research network. Interested GPs were purposively sampled in order to ensure the maximum possible variation in terms of their characteristics (sex and years of experience) and location (no more than two GPs from the same practice). A sample size of 20 was estimated based on Francis *et al*’s guidance for sample sizing of qualitative studies,^10^ but interviewing continued until saturation, defined as three consecutive interviews without additional themes identified.

### Interviews

Interviews each lasted 20–40 minutes and were conducted by telephone between November 2021 and June 2022 by an experienced qualitative researcher, using a flexible interview guide developed based on feedback from four GPs (see Supplementary Box S1 for details). Participants were asked to recall examples of past consultations with younger patients with cancer symptoms, discuss factors influencing their referral decisions, and relate their general interpretation and views on age thresholds in cancer referral guidelines. For four GPs, who were unable to provide an example of a past consultation to discuss, a vignette was used instead.

### Analysis

Anonymised verbatim transcripts of audiorecorded interviews were analysed using thematic analysis guided by a framework approach,^11^ under the oversight of an experienced qualitative researcher. This included familiarisation with the transcripts, iterative development of codes, coding of the transcripts, and identification of dominant themes and subthemes. Details of the coding and thematic framework are reported in Supplementary Table S1. Data were coded, organised, and summarised into framework matrices using NVivo software (version 1.6.1). All transcripts were coded by one researcher, with a random sample of 25% independently indexed by another researcher, to ensure consistency and reproducibility in the interpretation and application of the coding framework. Final interpretation of the data involved identifying the links between the themes and relating these findings back to the research questions and relevant literature. The researchers kept a reflective diary throughout data collection and analysis.

## Results

### Sample characteristics

Twenty-three GPs were interviewed, from 20 different practices. While a variety of recruitment strategies were used, the most successful was advertising through the Yorkshire and Humber Clinical Research Network. As a result, most GPs (*n* = 17) were based in Yorkshire. Fourteen GPs were male and 9 were female. Time working as a GP varied from 1 to 28 years (mean = 14 years, standard deviation = 8 years). Most GPs were of white ethnic background (*n* = 17) and worked in towns or cities (*n* = 15). Three GPs worked in multiple surgeries in both urban and rural locations. Six GPs had a research, professional, or personal interest in cancer and 4 covered roles within cancer alliances. GP and practice characteristics are summarised in Table 1.

**Table 1.** Characteristics of the GPs and practices included in the study

**Variable**		**Number**
GP sex	Male	14
	Female	9

GP ethnicity	White	17
	Asian	4
	Black	1
	Other	1

GP age, years	25–34	2
	35–44	8
	45–54	12
	≥55	1

Number of years of experience as GP	0–4	1
	5–9	7
	10–14	3
	15–19	6
	≥20	6

GP with specialist expertise in cancer	Yes	10
	No	13

Practice location	Yorkshire	
	*West Yorkshire*	8
	*North Yorkshire*	6
	*South Yorkshire*	3
	Greater London	3
	Cambridgeshire	1
	Tyne and Wear	1
	Leicestershire	1

Practice type	Urban	15
	Semi-rural	4
	Rural	1
	Mixture	3

### Thematic analysis

GPs mentioned a wide range of factors impacting their clinical decisions for younger people presenting with possible cancer symptoms. Analysis identified six main themes outlined below. See Supplementary Tables S2–S7 for details of further supporting quotes for each theme.

### Impact of patient age on GPs’ suspicion of cancer

GPs explained that a patient’s age had a strong influence on their referral decisions, as their suspicion of cancer was often lower in younger patients and they considered alternative explanations for symptoms first:

*‘It’s much harder to refer somebody younger, that your suspicion will be much lower, purely because of their age, even though you still might be concerned that*

*there is something wrong, you are more likely to think that it’s something less serious and therefore it doesn’t matter if they wait for a long time on a pathway.*’

(GP14)

The likelihood of an urgent cancer referral was said to be higher for cancers perceived as more common in younger people, or for which GPs had past experiences of a diagnosis in a younger patient. It was also influenced by ‘*how far off the qualifying age they would be for a cancer referral*’ (GP08):

‘*So, definitely for breast cancer is something that I don’t take the age into account very much and sometimes patients who have got GI* [gastrointestinal] *symptoms* [...] *I tend to be a bit less strict with the age referrals just because again I know that bowel cancer is something that is getting commoner and it is affecting younger people.*’

(GP01)

### Influence of clinical, patient-and GP-related factors in referral decisions

Influence of age on referral decisions was modulated by clinical factors, patient-related factors, and GPs’ professional and personal experiences.

With clear red flag symptoms, most GPs said they would refer patients urgently regardless of age. Urgent referral was also often used when findings of physical examinations, tests, or investigations were either concerning or inconclusive:

‘*If they’ve lost weight, they’ve got rectal bleeding and their bowels have changed, whether they are 30 or not I’m going to refer them as a fast track anyway.’* (GP05)

‘*When you’ve already got an imaging finding that it’s flagged up as suspicious, then I guess they’re confident that something’s not going to get rejected, purely on age.*’

(GP14)

Some GPs reported that patients’ characteristics, such as a family history of cancer, smoking, or ethnicity, influenced the perceived risk of some cancers and could therefore edge them towards referring younger patients urgently. Some GPs mentioned taking into consideration patients’ and relatives’ concerns and preferences, but these factors on their own were not usually sufficient to sway them towards an urgent referral:

‘*If they were very, very worried about cancer I could be swayed to take a more urgent referral approach than I would as myself maybe. I’m not sure whether that would make me refer on a 2-week wait pathway but it might make me do an advice and guidance referral or do some urgent blood tests or something if they were very worried about cancer.’*

(GP03)

GPs’ personal and professional experience of cancer increased their propensity to refer younger patients:

‘*I have a very low threshold to refer people just because of my own personal experiences … I didn’t tick any of the boxes and yet I still ended up getting cancer.*’

(GP01)

‘*I think we tend to be ruled sometimes by our previous experiences with patients, and we had a very sad case where actually a member of staff died from … cancer and they didn’t fit the criteria.*’

(GP22)

A special interest in cancer increased suspicion, confidence in referring, and ability to navigate the system:

‘*Being a cancer GP, my level of confidence is a lot higher and I feel able to navigate the system and advocate.*’

(GP09)

Several GPs mentioned ‘gut feeling’, which they describe as a ‘*sixth sense*’ (GP12) or a feeling that ‘*something doesn’t feel right*’ (GP06), as a crucial factor that may persuade them to refer younger patients urgently:

‘*Sometimes people come in walking through the door and they just look dreadful and you just kind of know there’s something wrong.*’

(GP20)

### GPs’ interpretation, application, and views of age criteria in cancer guidelines

There was some disagreement between GPs on the perceived flexibility of the guidelines, with some interpreting them as guidance and others as rigid rules. Some GPs were unsure about whether age criteria were meant to be interpreted flexibly, and suggested that this should be made clearer. As a result, some GPs applied the guidelines meticulously and referred ‘under age’ only very rarely, while others based their decisions on their own clinical judgement only:

‘*I don’t think there’s any flexibility in the cut-off age. But I’m hoping that the cut-off age is there for a good reason. The ones that have a cut-off age, I’m hoping that that’s been defined from good evidence* … *I personally treat it as a firm cut-off*’.’

(GP20)

‘*It does say in all the guidelines that you can refer outside the guidelines … maybe it needs to be more explicit in the guidelines because I don’t think it explicitly says outside the age range. Then I guess that might be interpreted differently by different people* … *I treat it as a guide, but as a fairly strong guide.*’

(GP14)

GPs held both positive and negative views of age criteria in cancer guidelines. While some GPs valued the guidance and understood its rationale, others found that sometimes it ‘*makes things a little bit awkward*’ (GP01) and they thought age criteria ‘*a bit prescriptive*’ (GP01). Some GPs believed that ‘*there should be a little bit more flexibility*’ (GP13) and one suggested that there should be no age criteria at all. Another GP questioned whether age criteria are applicable in areas of high deprivation because ‘*morbidity comes on much earlier*’

(GP15).

‘*There has to be some guidance around it because otherwise you would investigate everybody all the time and that’s not appropriate either and could be harmful to patients to do that.*’ (GP02)

‘*I think the age cut-offs probably need to go full stop and they need to have it as a little bit more of a clinician-led decision, because at the end of the day, GPs have also done a lot of training.*’ (GP01)

### Options and constraints for the referral of younger patients

GPs listed several options for investigation and referral of patients whose age falls below the age criteria, with some local variability. With high clinical concern, many GPs felt able to refer using the USC pathway, regardless of age:

‘*If I really thought that they had cancer there’s no barrier to me referring them urgently with suspected cancer, I would just have to explain what my concern was.*’

(GP02)

However, others said that they were unable to refer urgently outside guidelines because of the inflexibility of the local online pro forma, with one GP defining it as ‘*a computer-say-no sort of situation*’ (GP23). To circumvent this problem, some GPs described resorting to *‘make the symptoms kind of fit*’ (GP12), *‘bend the truth’* (GP03), and *‘find a way round the form’* (GP23). Other GPs would contact secondary care either through local services (for example, ‘Advice and Guidance’ by Consultant Connect, a service NHS GPs can use to contact specialists for patient-related advice) or their personal connections, as a way to expedite referral or receive advice on the best course of action:

‘*Our hands are a bit tied in the way we are working, in that we can’t refer on a particular pathway if they don’t qualify, so it’s greyed out, so there’s no option there … If they are under the age threshold I cannot physically refer them because the box is greyed out, therefore, I have to go and get round it, as I said earlier, like calling a consultant, getting permission and then to go round the houses and get my secretary to call the 2-week wait office*.’ (GP08)

‘*The young people almost fall in that indecision area where you’re like I’m not always convinced it’s necessarily going to be a cancer, but I’m also not necessarily happy about them waiting for that length of time … So it puts you in quite a difficult situation where you’re balancing your risks in your mind in terms of “Right, what am I going to do with this person?” and I think sometimes, if you’re that worried about it you end up sort of fudging some of the referrals or seeing if there’s some other way that they can meet the criteria in a different aspect that actually gets them seen*.’ (GP23)

In some areas, patients with concerning symptoms but not meeting age criteria could also be referred to a rapid diagnostic centre:

‘*We’ve got a vague symptoms pathway locally that we can refer through, that’s actually really helpful because that’s the patients that don’t really meet one of the defined 2-week wait criteria according to our local systems, but one of the criteria you can use to refer a patient through the pathway is GP gut feeling that the patient has cancer, which is great.*’

(GP20)

With lower clinical concern, many GPs would choose less urgent referral routes or would investigate younger patients in primary care to either exclude cancer or justify a USC referral. However, some GPs explained that a local lack of access to investigations, long delays for test results, or waiting times for non-urgent referrals could persuade them to refer urgently despite the patient not meeting age criteria:

‘*For these patients who, if there’s some concern but they don’t quite meet the pathway for a 2-week wait referral, you can send them off one of these bowel blood tests, the FIT test* [faecal immunochemical test]*, and if that’s negative you can be more reassured and if it’s positive then you put them on the 2-week wait pathway.*’

(GP21)

‘*I know if I refer a 35-year-old who’s got worrying symptoms for an upper GI endoscopy routinely, it can 6 months plus, which is obviously ridiculous and that’s another factor for why I might want to fast track them to try and get an answer a lot sooner for the patient and myself.*’

(GP12)

### GPs’ experience of, and attitude towards, rejection and criticism of referrals outside age criteria

While many GPs had never had a referral rejected because of age, others had ‘*worked in some areas where it happens a lot*’ (GP03). Worrying about the additional delay caused by a rejection of a USC referral led some GPs to invest considerable time in justifying their clinical rationale in the referral form, or to opt for other types of referral or investigative routes:

‘*I always write a referral letter regardless but I probably, in anticipation of it getting rejected, just based on the age, I’d make sure I write more into the letter to explain my concerns. I don’t think I’ve had a patient rejected.*’

(GP18)

Several GPs explained that they had experienced personal criticism for referrals in the past, but they stated that this did not influence their decisions. While some GPs reported friction with secondary care regarding referrals, others described how a supportive relationship with staff at the local hospital had a positive impact on patient management:

‘*The frustrating ones are when they get rejected yet you’ve spent a long time filling out all the extra sections on the form as to why you think this person really needs to be seen even though they don’t strictly meet the box above. But if the box above still is not ticked, whoever’s in the office will still reject it and send it back.*’

(GP23)

‘*We’ve got a really good relationship with all our colleagues, and I think that they know that we’re not going to refer unnecessarily. Whether you’d get that in a big, if you went to a big teaching hospital, and you haven’t got that same relationship, it might be a little bit more awkward.*’

(GP19)

### Considering the consequences of referral outside age guidelines

GPs explained that their decisions were influenced by the perceived consequences of referral on both the individual patients and the health system as a whole. GPs were painfully aware of the possible adverse consequences of missing a cancer diagnosis, with some even recalling losing sleep over some decisions. However, GPs were also mindful of the harms of an unnecessary referral in younger patients at lower risk of cancer.

GPs were divided between their roles as gatekeeper and patient advocate. Some felt the need to ‘*think carefully about referring*’ (GP02) and ‘*act responsibly with resources*’ (GP04), to avoid causing delays for patients at higher risk of cancer and putting excessive burden on secondary care:

‘*If you refer too many younger people, then your higher risk patients who are older and at a higher risk of cancer are going to wait even longer for their tests, so it makes sense that it’s set up the way that it is.*’

(GP21)

However, others believed that their ‘*responsibility as a GP is to the patient more than to the wider public*’ (GP12) and that ‘*it’s not my job to ration stuff*’ (GP04) or ‘*trying to save money for the local health service*’ (GP12):

‘*I always see the guidance as trying to filter things so there’s not as much pressure on the system, which I can understand but that goes against the job that you do. I feel like my responsibility as a GP is to the patient more than to the wider public but I know that certain GPs are better at balancing that*.’

(GP12)

## Discussion

### Summary

This study captures GPs’ interpretation, application, and views of age criteria in cancer referral guidelines and explores the factors influencing their referral decisions for younger patients with symptoms of cancer. The data show variability in interpretation of age criteria, with some GPs perceiving and applying them more rigidly than others and a contrast between some areas, where GPs are empowered to refer urgently based on their ‘gut feeling’, and others where the local referral systems impose more rigidity. Although GPs understood the rationale behind age criteria in cancer guidelines, some found them restrictive and would have preferred guidelines that put more emphasis on the clinician’s own judgement. Reassuringly, in the presence of clearly concerning symptoms, most GPs would use the USC referral pathways regardless of a patient’s age. Other factors that made GPs more likely to consider a USC referral in younger patients were abnormal results of investigations, a lack of explanation and/or resolution for symptoms, a strong family history of cancer, GPs’ personal or professional experience of cancer diagnosis in younger patients, and a strong ‘gut feeling’ that the patient may have cancer. In contrast, less obvious symptoms or alternative explanations for the symptoms made GPs more likely to either investigate the patient in primary care or refer through non-urgent pathways. Considering the impact on system resources and finances, worrying about referral rejection, or inflexible online pro forma dissuaded some GPs from referring urgently outside age guidelines. Patient concerns and preference had only a limited bearing on decisions, while fear of criticism was said to have no effect. GPs reported a wide regional variability in the access to investigative options, rapid diagnostic centres, local clinics, and efficient non-urgent pathways, which had an impact on clinical decisions.

### Strengths and limitations

Strengths of the study include robust framework analysis and variation in terms of GP’ and practice’ characteristics. For instance, while some GPs had personal or professional interest in cancer, others did not, ensuring a range of views were captured in the study. A limitation of the study is that all GPs were based in England, with most being based in Yorkshire, therefore the data may not be representative of the whole of the UK. The interviews were conducted by telephone as this provides significant practical advantages compared with in-person interviews (for example, being easier to fit in to GPs’ busy schedules, and having the ability to recruit across a large geographical area) or video interviews (such as no need for a stable internet connection).^12^ While this choice may have resulted in missing non-verbal cues or reducing rapport, this is likely to have had a limited impact on the quality of the data, as the topic did not require disclosure of sensitive information and/or communication of emotions.

### Comparison with existing literature

Previous studies have explored GPs’ views and application of cancer referral guidelines in general.^13^^,^^14^ This study extends previous research by focusing on age criteria and, in particular, decision making surrounding younger patients. The findings add to earlier work demonstrating that, although GPs consider the guidelines helpful overall, especially for patients with clear-cut symptoms, they may act as a barrier for patients not fitting the criteria, because of the perceived lack of flexibility and limited scope for clinical judgement.^13^^–^15 Although the role of ‘gut feeling’ in driving GPs’ decisions has been examined previously,^16^^,^^17^ the findings of this study extend the literature on its crucial influence on referral of younger patients. GPs described a struggle in reconciling their dual role as patient advocate and gatekeeper of resources, and the deep sense of worry of potentially missing a cancer diagnosis, which often led them to a cautionary approach. This echoes previous studies,^13^^,^^14^ but is the first report in relation to the decisions around management of younger patients with possible symptoms of cancer. Many GPs described their concerns about long delays for non-urgent referrals or tests. GPs explained they often followed up patients to make sure they were investigated within a reasonable timeframe, used their local knowledge to find alternative diagnostic options, or resorted to urgent referral even when cancer suspicion was lower. Some also described how a supportive relationship between primary and secondary care was crucial in achieving a timely diagnosis in younger patients. This reinforces previously reported referral system barriers and strategies to overcome them through the positive influence of good relationships with colleagues in secondary care.^13^^,^^14^

### Implications for practice

The findings of this study highlight important opportunities to improve diagnosis of cancer among younger patients in primary care. There is a need for a more widespread understanding among GPs that, when there is sufficient clinical concern regarding cancer, current guidelines permit urgent referral outside age criteria. While NICE guidelines for suspected cancer already state that ‘it is not mandatory to apply the recommendations, and the guideline does not override the responsibility to make decisions appropriate to the circumstances of the individual,’^8^ their next iteration could present this statement more prominently and include it in the condensed guidelines for each cancer type, which clinicians are more likely to refer to on a routine basis. While GPs must retain the responsibility to steward resources effectively, they should also be supported in applying their clinical judgement, as envisaged in the guidelines. Therefore, awareness needs to be raised in regional health systems that local implementation of USC pathways should, when suspicion of cancer is significantly high, permit referral of those who do not meet specific age criteria. To this end, in some areas local referral forms need to be altered so that they do not impose undue barriers to referral solely based on a patient’s age.
